# Impact of sarcopenia and myosteatosis on survival outcomes for patients with head and neck cancer undergoing curative-intent treatment

**DOI:** 10.1017/S0007114522000435

**Published:** 2023-02-14

**Authors:** Elizabeth Ahern, Teresa Ellen Brown, Louise Campbell, Brett G. M. Hughes, Merrilyn Banks, Charles Y. Lin, Lizbeth M. Kenny, Judith Bauer

**Affiliations:** 1 Medical Oncology, Monash Health, Clayton, VIC 3168, Australia; 2 School of Medicine, Monash University, Clayton, VIC 3168, Australia; 3 School of Human Movement and Nutrition Sciences, University of Queensland, St Lucia, QLD 4072, Australia; 4 Department of Nutrition and Dietetics, Royal Brisbane and Women’s Hospital, Herston, QLD 4029, Australia; 5 Department of Nuclear Medicine and Specialised PET Services Queensland, Royal Brisbane and Women’s Hospital, Herston, QLD 4029, Australia; 6 Cancer Care Services, Royal Brisbane and Women’s Hospital, Herston, QLD 4029, Australia; 7 School of Medicine, University of Queensland, Herston, QLD 4029, Australia; 8 QIMR Berghofer Medical Research Institute, Herston, QLD 4029, Australia

**Keywords:** Head and neck cancer, Sarcopenia, Myosteatosis, Nutrition support, Enteral feeding

## Abstract

Malnutrition and sarcopenia are prevalent in patients with head and neck squamous cell carcinoma (HNSCC). Pre-treatment sarcopenia and adverse oncological outcomes in this population are well described. The impact of myosteatosis and post-treatment sarcopenia is less well known. Patients with HNSCC (*n =* 125) undergoing chemoradiotherapy, radiotherapy alone and/or surgery were assessed for sarcopenia and myosteatosis, using cross-sectional computed tomography (CT) imaging at the third lumbar (L3) vertebra, at baseline and 3 months post-treatment. Outcomes were overall survival (OS) at 12 months and 5 years post-treatment. One hundred and one participants had a CT scan evaluable at one or two time points, of which sixty-seven (66 %) participants were sarcopenic on at least one time point. Reduced muscle attenuation affected 93 % (*n* = 92) pre-treatment compared with 97 % (*n* = 90) post-treatment. Five-year OS favoured those without post-treatment sarcopenia (hazard ratio, HR 0·37, 95 % CI 0·16, 0·88, *P* = 0·06) and those without both post-treatment myosteatosis and sarcopenia (HR 0·33, 95 % CI 0·13, 0·83, *P* = 0·06). Overall, rates of myosteatosis were high at both pre- and post-treatment time points. Post-treatment sarcopenia was associated with worse 5-year OS, as was post-treatment sarcopenia in those who had myosteatosis. Post-treatment sarcopenia should be evaluated as an independent risk factor for decreased long-term survival post-treatment containing radiotherapy (RT) for HNSCC.

Malnutrition is prevalent in up to 50 % of patients with head and neck squamous cell cancer (HNSCC), owing to tumour factors (such as anatomic location and metabolic requirements) and treatment factors (such as acute and chronic toxicities of radiotherapy, chemotherapy or surgery) reducing oral intake^(1,2)^. Malnutrition associated with cancer comprises loss of skeletal muscle with or without loss of adipose tissue, associated with weight loss^(3,4)^. Sarcopenia is a term with heterogenous definitions and applications, but which expert consensus has defined as a combination of skeletal muscle depletion combined with functional impairment^(5)^. A related but distinct entity associated with cancer is myosteatosis, which relates to intramuscular adipose infiltration, detected on cross-sectional imaging as a reduction in muscle tissue density^(6)^. Both sarcopenia and myosteatosis have been associated with adverse outcomes in various cancers, including excess mortality^(6–8)^. Heterogeneity in the literature arises in the definition and assessment of sarcopenia and body composition radiologically, which requires methodological consensus^(9)^. A frequent method comprises assessment of a cross-sectional area on computed tomography (CT) scan (or the CT component of positron emission tomography (PET) scan) at the level of the third lumbar vertebra (L3) where a reference sex-specific lower limit of normal skeletal muscle index (SMI) is applied^(10–14)^.

Sarcopenia may be primary (age-related) or secondary (activity, disease such as cancer or nutrition-related)^(15,16)^. Sarcopenia, when defined pragmatically in largely retrospective trials as low radiologically assessed SMI, has been associated with excess chemotherapy toxicity, increased postoperative complications and decreased overall survival (OS) in patients with HNSCC of various stages^(10–13,17–20)^. When accompanied by systemic inflammation, sarcopenia in patients treated with definitive radiotherapy or chemoradiotherapy (CRT) for HNSCC was most correlated with decreased overall and progression-free survival on multivariate analysis and was also associated with radiotherapy interruptions^(14)^. Other adverse outcomes have been associated with baseline sarcopenia in some studies, such as prolonged feeding tube dependency^(21)^. Certain demographic subsets may be at excess risk of sarcopenia: in elderly patients with HNSCC (aged 70 years and above), over 80 % had low baseline skeletal muscle mass, and where this was accompanied by low muscle function, sarcopenic patients had significantly reduced OS^(22)^. In one study of HNSCC patients, women had a significantly lower median baseline SMI despite having similar baseline BMI^(23)^, although whether this represents a risk for development of sarcopenia during therapy was not explored. Although low BMI was correlated elsewhere with sarcopenia in HNSCC patients, BMI itself or absolute weight loss during therapy does not appear to account for the significant associations noted between sarcopenia and impaired survival outcomes in HNSCC^(13)^. Furthermore, in addition to baseline sarcopenia, reduced OS has been associated with a differential finding of skeletal muscle depletion during radiotherapy, which was also associated with impaired locoregional cancer control^(12)^. Whether post-treatment sarcopenia is similarly associated with impaired outcomes is less well described but similarly appears to be associated with decreased survival^(11,12)^. Similarly, the significance of pre- or post-treatment myosteatosis on oncological outcomes in the HNSCC population is unclear.

Sarcopenia in the context of cancer comprises a category of diagnosis of cancer cachexia, defined by an ongoing loss of skeletal muscle mass with or without loss of fat mass, which is a potentially multifactorial entity associated with reduced functioning and impaired cancer outcomes^(3)^. Cancer-associated cachexia and weight loss are generally suboptimally assessed and managed^(24,25)^, despite oncology-specific nutrition guidelines including in HNSCC populations^(26–29)^. Although management of cachexia is complicated by its multifactorial aetiology and likely requires a multidisciplinary approach^(2)^, patients with HNSCC may have a more favourable response to nutrition support due to the alleviation of mechanical obstruction which may have been a major contributing factor to pre-diagnosis skeletal muscle depletion and weight loss. Whether proactively supporting nutrition of HNSCC patients, for example, via early institution of supplementary nutrition, prevents weight loss and sarcopenia development while on treatment remains unclear, although selective prophylactic gastrostomy insertion has been shown to improve nutrition outcomes^(30)^. A prior study showed no significant differences in weight loss outcomes (following intention-to-treat analysis) when comparing early-intervention gastrostomy feeding with standard care in patients treated with curative intent for HNSCC; however, poor participant adherence with the early intervention measures was a likely major confounder^(31)^. Here, we extend the results of this study to a radiological assessment of baseline and post-treatment sarcopenia and myosteatosis in this patient population, derived from fluorodeoxyglucose-PET/CT (FDG-PET/CT) scans performed as part of standard care. We further assess the impact of sarcopenia and myosteatosis on OS outcomes at 12 months and 5 years.

## Methods

### Study participants and interventions

This is an observational study and a priori secondary analysis of patients who participated in a prospective randomised controlled trial. The trial protocol and primary outcomes have been previously published^(31,32)^ and reported using The Consolidated Standards of Reporting Trials (CONSORT) statement.

To briefly summarise, 131 patients were included as part of a single-institution, parallel-group, randomised controlled trial conducted at the Royal Brisbane and Women’s Hospital (Queensland, Australia). Eligible patients were identified through the head and neck multidisciplinary clinic as those treated with curative intent for HNSCC who were referred by the treating team for prophylactic gastrostomy. Exclusion criteria included non-curative-intent treatment or pre-existing moderate/severe malnourishment or significant dysphagia requiring a modified diet. Definitive or adjuvant CRT was received by 94 % of participants, with the remainder receiving either definitive or adjuvant radiotherapy (RT) alone or surgery. One hundred and thirty-one participants were recruited and randomised 1:1 into one of two arms (early nutrition intervention *v*. standard care), stratified by baseline nutritional status (well nourished or malnourished). The study intervention comprised either early nutrition intervention (initiation of enteral nutrition via gastrostomy immediately following prophylactic tube placement) or standard care (initiation of enteral nutrition based on clinical indicators reflecting insufficient oral intake and/or need for modified diet). Nutritional status was assessed using the Patient-Generated Subjective Global Assessment tool (PGSGA), and weight, BMI and body composition (using bioelectrical impedance analysis) were also all measured at baseline and at 3 months post-treatment completion. Baseline nutrition characteristics were balanced between groups; 76 % were PGSGA category A (well nourished); median PGSGA risk score was 6 and median BMI at baseline was 27·2 kg/m^2^, and these nutrition outcomes post-treatment have been fully described previously^(31)^.

### Ethical approval

The study was approved by the Human Research Ethics Committees of the Royal Brisbane and Women’s Hospital on 19 July 2012 (HREC/12/QRBW/162) and The University of Queensland on 8 August 2012 (2012000890) and registered on the Australian New Zealand Clinical Trials Registry (ACTRN12612000579897).

### Survival outcomes

Treatment response was assessed via FDG-PET/CT scan at approximately 3 months post-treatment completion. Participants were followed up for survival after completion of anti-cancer treatment. Survival outcomes were assessed at 12 months and 5 years post-treatment completion and time (in months) to documented cancer relapse and/or death was recorded. OS was defined as time in months between completion of anti-cancer treatment and death from any cause or last follow-up.

### Assessment of sarcopenia and myosteatosis

A single observer (JB) evaluated CT images for sarcopenia and myosteatosis. This observer was blinded to participants’ outcomes and trained in CT analysis. Sarcopenia was derived from FDG-PET/CT scan L3 tissue density data with muscle (–29 to +150 Hounsfield units (HU)) quantified using Slice-O-Matic software (version 5.0, TomoVision). Sarcopenia was defined as SMI < 41 cm^2^/m^2^ (females) and <43 cm^2^/m^2^ (males) in underweight or healthy weight range participants (BMI ≤ 24·9 kg/m^2^) or <53 cm^2^/m^2^ in overweight or obese participants (BMI ≥ 25 kg/m^2^)^(8)^. Those with a SMI above these sex-defined levels were deemed non-sarcopenic. Myosteatosis was assessed through calculation of mean muscle attenuation on CT scan (MACT) for the entire L3 muscle area. Myosteatosis was defined for both sexes as those with low MACT, according to BMI as follows: BMI ≤ 24·9 kg/m^2^, mean MACT < 41 HU, and BMI ≥ 25 kg/m^2^, mean MACT < 33 HU^(8,33)^. FDG-PET/CT scans were conducted at two time points, baseline (pre-treatment) and to assess treatment response (approximately 3 months post-treatment), according to routine clinical practice.

### Statistical methods

As this was a secondary analysis, a power calculation was not appropriate, as sample size was dictated by recruitment in the prior study. For multivariable analysis, analyses were performed on the intention-to-treat population with participants considered sarcopenic if they were assessed as sarcopenic at either time point. Categorical variables were summarised using frequency and percentage and continuous variables by mean and standard deviation for normally distributed variables or median and interquartile range (IQR) for non-normally distributed variables. Univariate associations between sarcopenia status and categorical patient characteristics (age, stage according to AJCC 8th edition, P16 status via immunohistochemistry, sex, smoking status, T-score of primary tumour, diet, nutritional status and intervention group assigned to in the randomised controlled trial) were examined using χ^2^ tests of independence or the Fisher’s exact test, where more than 20 % of the expected values were less than 5. Association between age and sarcopenia status was examined using a two-sample *t* test, with variance equality assessed using Levene’s test.

A logistic regression model was run which included all variable found to be associated with sarcopenia status at the 20 % significance level in univariate analyses. A backwards stepwise approach was used, with the Wald statistic as a cut-off for inclusion in the final logistic regression model at *α* < 0·05. For categorical measures with greater than two groups, categories were combined as appropriate for insufficient numbers or for imbalanced categories. For column statistics comparing two groups, comparison was by unpaired *t* test except in the situation of unequal variances, where Mann–Whitney U-test was then used. For column statistics comparing three or more groups, with equal variances, one-way ANOVA with Tukey’s post-test analysis for multiple comparisons was used. In the event of unequal variances reflected by a significant (*P* < 0·05) Bartlett’s test, Kruskal–Wallis method was used to compare three or more groups with Tukey’s post-test for multiple comparisons.

For survival, a landmark analysis was undertaken with OS calculated from the date of the post-treatment PET-CT scan. The Kaplan–Meier method was used to compare OS between various groups at 12 months and 60 months post-conclusion of CRT or RT, with difference in curves assessed by log-rank method for hazard ratio (HR). Ninety-five per cent CI for HR was also calculated. Where more than two groups were compared using Kaplan–Meier technique, differences between curves were assessed using Mantel–Cox test. For all statistical analyses, significance (*α*) was deemed *P* < 0·05 (two-sided).

## Results

### Patient characteristics

Of the initial 131 participants reported in the previous study, six subsequently withdrew consent and have been excluded, leaving 125 participants assessed for outcomes in this study. Of these remaining participants 97 (78 %) had an oropharyngeal primary, of which 85 (88 % of oropharyngeal group) were P16-positive. Included patients had disease of various stages, from I-IVA as per AJCC 8th edition, but all were treated with curative intent. Most patients (94 %) received definitive (*n* = 109) or adjuvant (*n* = 8) CRT. Five patients received postoperative radiotherapy (4 %), one received radiotherapy alone (1 %) and two received surgery alone (1 %). Mean age (±sd) was 60·5 years (±10·1 years), and 88 % were male.

### Sarcopenia prevalence and characteristics

**Fig. 1.**
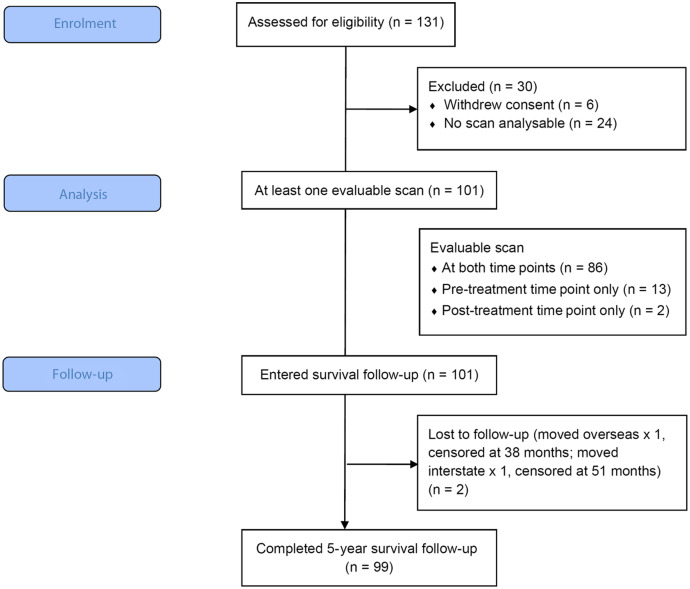
CONSORT diagram for study participants.

Although 125 participants were assessed for outcomes in the study, 24 lacked an analysable scan for sarcopenia assessment at both time points (Fig. 1). Median time between scans was 181 (IQR 174·5–195·5) days. Of the 101 participants with at least one time point analysable for sarcopenic status, the prevalence of sarcopenia was 66 % (67 participants). Table 1 summarises clinicopathological characteristics in the total analysable population and between those with and without sarcopenia. Only age was found to be associated with sarcopenia status in univariate analysis (*P* < 0·001) at the *α* < 0·05 significance level; however, AJCC 8th edition stage and P16 status were included in model building at the *α* < 0·20 significance level. The final logistic regression model is displayed in Table 2, with only age found to be associated with sarcopenia status. Results of the model indicate there is strong evidence to suggest the odds of sarcopenia development increases by 9 % (95 % CI 4 %, 15 %, *P* < 0·001) for each 1-year increase in age.

**Table 1.** Baseline clinicopathological characteristics overall and between sarcopenia status groups (Number and percentages)

	Overall (*n* = 101)		Not sarcopenic (*n* = 34)		Sarcopenic (*n* = 67)		*P*
	*n*	%	*n*	%	*n*	%	
Sex							1·00
Male	92	91·1 %	31	91·2 %	61	91·0 %	
Female	9	8·9 %	3	8·8 %	6	9·0 %	
Age	61·2	9·7	56·5	9·3	63·6	9·1	<0·001
Smoking Status							0·92
Non-smoker	23	22·8 %	8	23·5 %	15	22·4 %	
Ex-smoker	62	61·4 %	20	58·8 %	42	62·7 %	
Current smoker	16	15·8 %	6	17·6 %	10	14·9 %	
Stage (AJCC 8th)							–
I	23	22·8 %	12	35·3 %	11	16·4 %	
II	35	34·7 %	13	38·2 %	22	32·8 %	
III	25	24·8 %	5	14·7 %	20	29·9 %	
IVA	18	17·8 %	4	11·8 %	14	20·9 %	
Stage (categorised)							0·032
I or II	58	57·4 %	25	73·5 %	33	49·3 %	
III or IVA	43	42·6 %	13	26·5 %	34	50·7 %	
T-score							0·51
T0/T1	42	41·6 %	16	47·1 %	26	38·8 %	
T2	31	30·7 %	11	32·4 %	20	29·9 %	
T3/T4	28	27·7 %	7	20·6 %	21	31·3 %	
P16 status							0·19
Positive	78	79·6 %	30	88·2 %	48	75·0 %	
Negative	20	20·4 %	4	11·8 %	16	25·0 %	
Diet							0·25
Full	72	71·3 %	27	79·4 %	45	67·2 %	
Modified Texture	29	28·7 %	7	20·6 %	22	32·8 %	
Nutritional status (*n* = 99)							
Well nourished	80	80·8 %	36	78·3 %	44	83·0 %	0·61
Malnourished	19	19·2 %	10	21·7 %	9	17·0 %	
RCT assigned group*							0·40
Standard	53	52·5 %	20	58·8 %	33	49·3 %	
Intervention	48	47·5 %	14	41·2 %	34	50·7 %	

RCT, randomised controlled trial.

* All patients had a prophylactic gastrostomy placed prior to treatment. Patients in the standard care group commenced nutrition support when clinically indicated. Patients in the intervention group commenced nutrition support prophylactically in addition to oral intake prior to treatment commencement.

**Table 2.** Logistic regression model for predictors of sarcopenia (Odds ratio and 95 % confidence intervals)

Model (*n* = 101)	Odds ratio	95 % CI	*P*	–2 Log-likelihood
Age*	1·09	1·04, 1·15	0·001	116·1
Constant†	2·17	1·38, 3·41	0·001	

* Centred.

† Baseline odds are shown for the model intercept at mean age of 61·2 years (not OR).

Eighty-six participants had an analysable scan at both time points (baseline/pre-treatment and post-treatment). Of these, forty-three (50 %) were sarcopenic at both time points, whereas twenty-four (28 %) were non-sarcopenic at both time points; twelve (14 %) were non-sarcopenic pre-treatment but were sarcopenic post-treatment and seven (8 %) were sarcopenic pre-treatment but non-sarcopenic post-treatment. At baseline, 53 of 99 (54 %) participants were sarcopenic, while 57 of 88 (65 %) participants with a post-treatment assessment were sarcopenic. Eighty-one per cent (*n* = 43/53) of participants who were sarcopenic at baseline remained sarcopenic, while 13 % (*n* = 7) of those became non-sarcopenic. Thirteen participants had CT scan available at baseline only (no post-treatment scan or unanalysable), and of these, ten were not sarcopenic but three were sarcopenic. Two had post-treatment scan only (no baseline) and were both sarcopenic at that time point.

At baseline, nineteen (19 %) participants were malnourished with nine of these sarcopenic. There was no difference in PGSGA score between sarcopenic (6·8) and non-sarcopenic (5·7) participants (*P* = 0·267).

### Myosteatosis prevalence

At baseline, ninety-nine participants had MACT and ninety-two (93 %) had muscle attenuation values consistent with myosteatosis; of these, fifty-one (55 %) were also sarcopenic. Of the seven patients who had normal MACT at baseline, five had MACT assessed also post-treatment. Two (29 %) had sarcopenia despite normal MACT at both time points, whereas three had normal MACT and no sarcopenia at both time points. Of the remainder, four of the ninety-two (4 %) with low MACT at baseline had normal muscle density post-treatment but eighty-eight of ninety-two (96 %) continued to have low MACT post-treatment. Two additional participants had low MACT post-treatment, but they did not have a baseline assessable scan. Overall, three of eighty-nine (3 %) of post-treatment scans displayed normal MACT.

### Sarcopenia and myosteatosis association with BMI and weight change

**Fig. 2.**
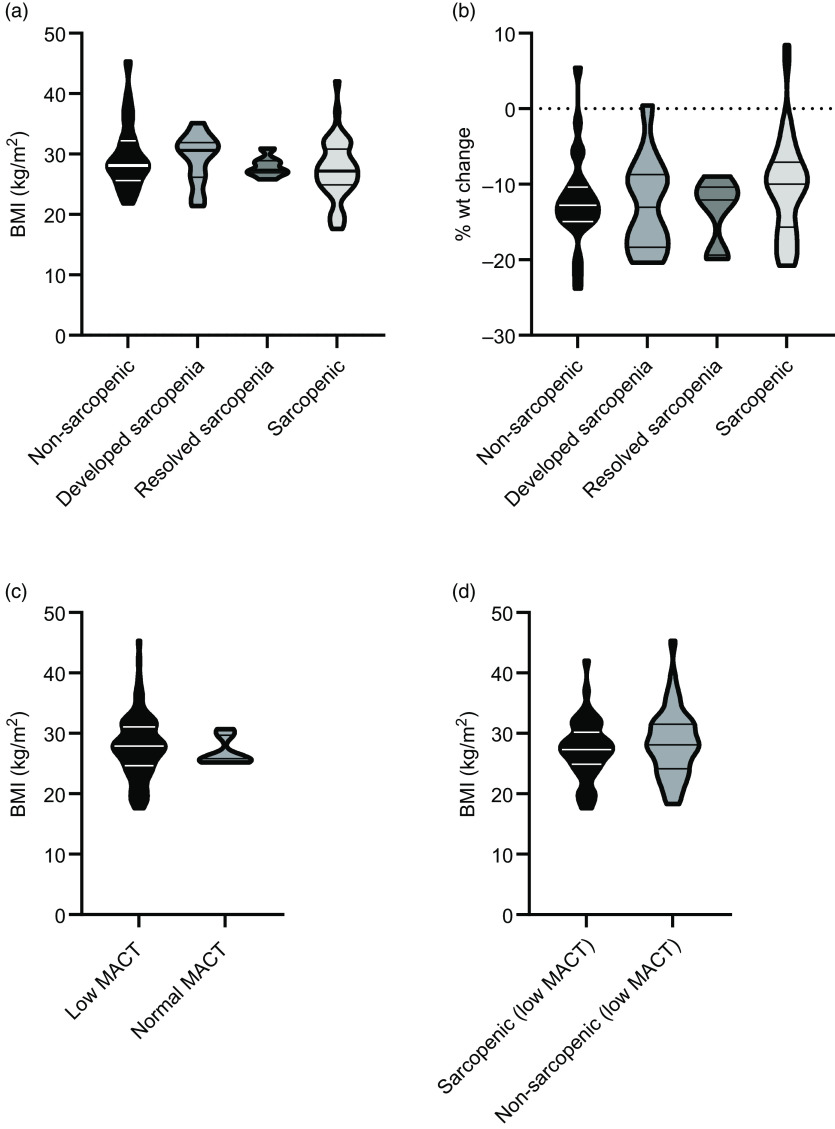
Association between sarcopenia and myosteatosis status BMI over the course of treatment. No significant difference was seen with respect to (a) BMI when assessed pre-treatment (chemoradiotherapy) or (b) percentage weight (wt) change over the course of the treatment when considering participants who were non-sarcopenic at both pre- and post-treatment time points (‘non-sarcopenic’), those who were sarcopenic at both time points (‘sarcopenic’), those who were non-sarcopenic at pre-treatment but sarcopenic post-treatment (‘developed sarcopenia’) or vice versa (‘resolved sarcopenia’). Similarly, no significant difference was seen in BMI when comparing groups who had (c) myosteatosis as reflected as low muscle attenuation on CT (MACT) and those with normal MACT, or (d) low MACT and sarcopenia compared with low MACT and no sarcopenia, when assessed at pre-treatment scan. Violin plots with lines indicating median and interquartile ranges.

BMI at pre-treatment and percent weight change comparing pre-treatment (baseline) and post-treatment was assessed for the following four groups: those who were non-sarcopenic at both time points (‘non-sarcopenic’), those that were sarcopenic at both time points (‘sarcopenic’), and those who changed category: from non-sarcopenic at baseline to sarcopenic post-treatment (‘developed sarcopenia’), and vice versa (‘resolved sarcopenia’). There was no significant difference between the groups for either variable (Fig. 2(a) and (b)). Median weight change per group ranged from –10 % to –13 %, whereas median BMI ranged from 27·2 to 30·6 per group. Similarly, BMI at baseline was assessed for those who had low MACT and those who had normal MACT at pre-treatment, and there was no significant difference, with median BMI 27·9 in the low MACT group compared with 25·8 in the normal MACT group (Fig. 2(c)). BMI was also not significantly different at baseline between those who had low pre-treatment MACT and were sarcopenic (median 27·3) compared with not sarcopenic (median 28·1) (Fig. 2(d)).

### Sarcopenia and myosteatosis association with survival outcomes

**Fig. 3.**
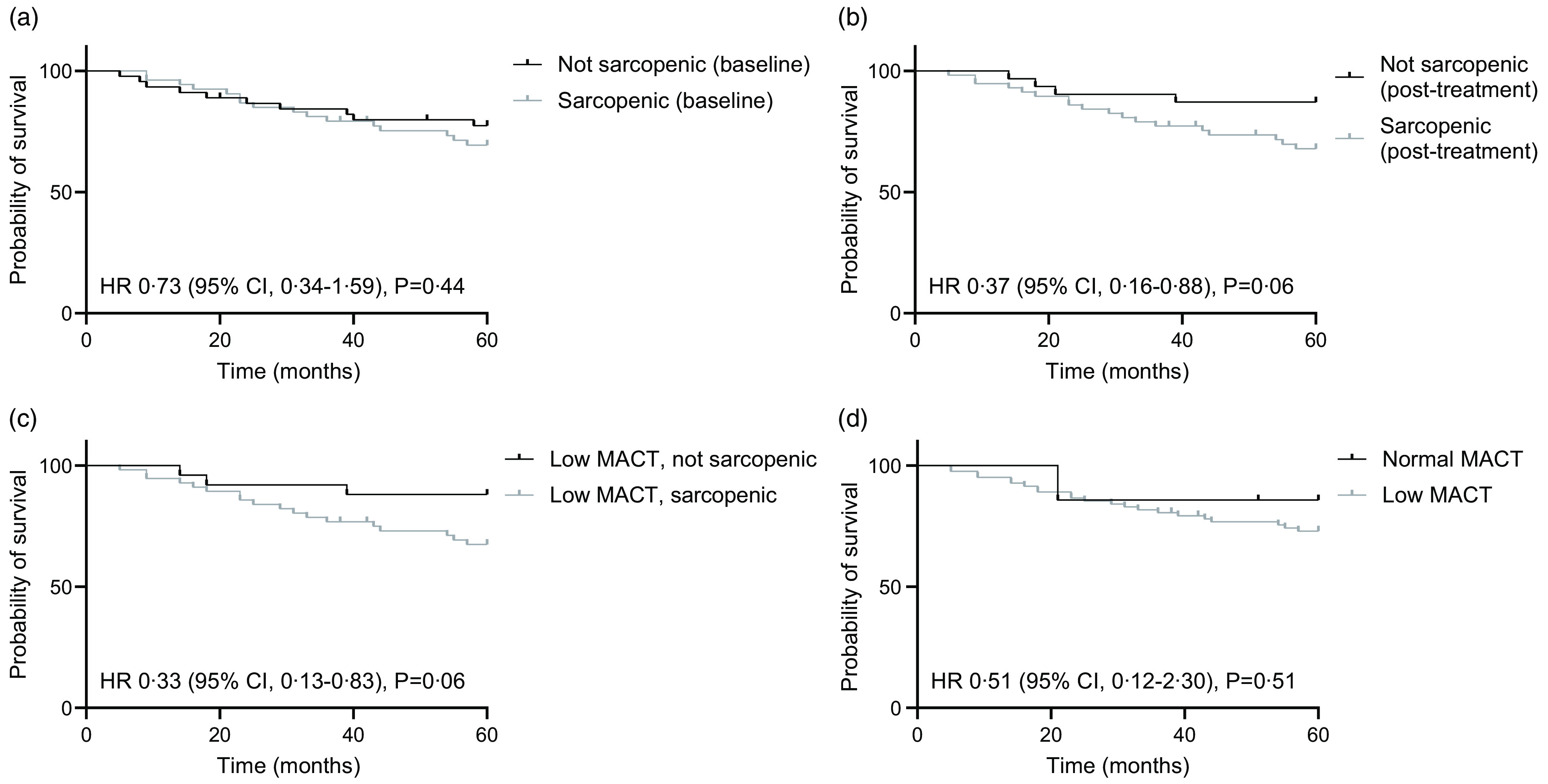
Survival outcomes according to sarcopenia and myosteatosis status. Overall survival at 60 months post-completion of chemoradiotherapy was assessed for (a) participants who were sarcopenic or not when assessed on pre-treatment scan, (b) participants who were sarcopenic or not when assessed on post-treatment scan, (c) in the subgroup of participants with myosteatosis reflected as low muscle attenuation on CT (MACT), those who were additionally sarcopenic or not, when assessed at post-treatment scan, and (d) those participants with myosteatosis reflected as low MACT or not on post-treatment scan. Hazard ratio (HR) (log-rank) with 95 % CI and *P*-value (log-rank, Mantel–Cox) displayed for each.

OS was assessed at two time points: 12 months (1 year) and 60 months (5 years) post-conclusion of HNSCC treatment. No significant difference was seen when comparing 1-year OS on the basis of sarcopenia status or myosteatosis when assessed at either pre-treatment or post-treatment time points (data not shown). Sarcopenia, when assessed pre-treatment, was also not significantly associated with 5-year OS (Fig. 3(a)). In contrast, the HR for 5-year OS favoured the non-sarcopenic group when assessed post-treatment (HR 0·37, 95 % CI 0·16, 0·88, *P* = 0·06) (Fig. 3(b)). When this analysis was further enriched by considering only those with low post-treatment MACT, the group without both sarcopenia and low MACT post-treatment showed better 5-year survival (HR 0·33, 95 % CI 0·13, 0·83, *P* = 0·06) (Fig. 3(c)) but with a similar HR to that seen with non-sarcopenic participants alone regardless of MACT assessment. Low MACT alone was not significantly associated with different 5-year OS when compared with those with normal MACT (Fig. 3(d)).

**Fig. 4.**
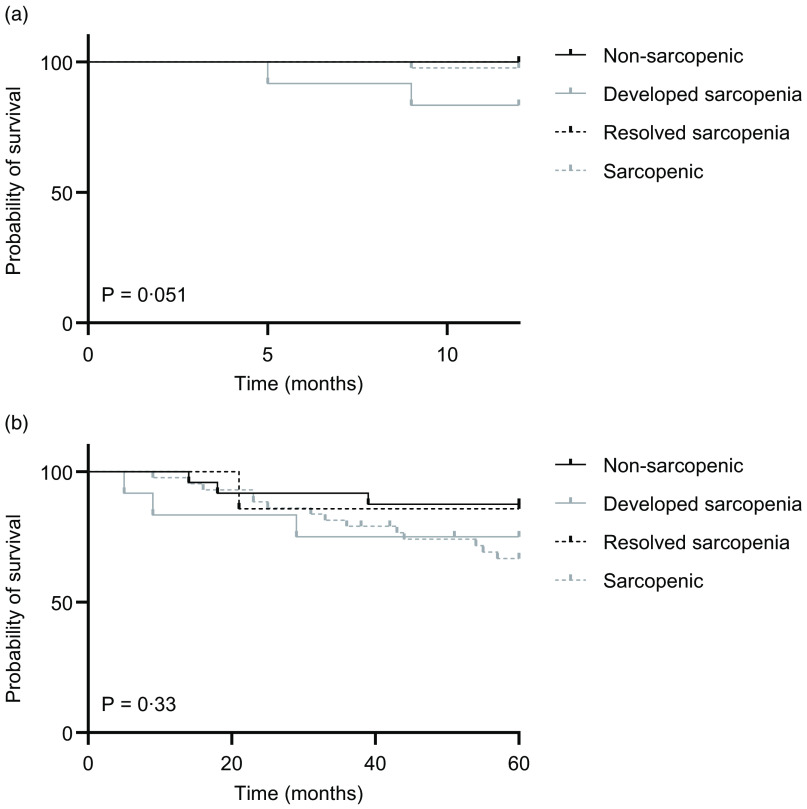
Overall survival according to sarcopenia status. Kaplan–Meier curves for OS at (a) 1 year and (b) 5 years for the four groups as described in Fig. 2(a) and (b). Log-rank (Mantel–Cox) test used to generate *P*-values.

Differential survival outcomes were further assessed when comparing those who were non-sarcopenic at both baseline and post-treatment time points (‘non-sarcopenic’), those that were sarcopenic at both time points (‘sarcopenic’), and those who changed category: from non-sarcopenic at baseline to sarcopenic post-treatment (‘developed sarcopenia’), and vice versa (‘resolved sarcopenia’). Although a trend towards worse OS for those who developed sarcopenia was evident at 12 months (*P* = 0·051), no significant difference was seen at 5 years (*P* = 0·33) (Fig. 4(a) and (b)).

## Discussion

This study contributes to the increasing literature and evidence relating to the impact of sarcopenia (using radiological criteria) on survival outcomes^(34)^. In this study, sarcopenia is assessed, at both pre-treatment and post-treatment time points, for its impact upon survival outcomes in a cohort of patients treated with curative intent for HNSCC. In addition, this is one of the few studies which also considers the assessment of myosteatosis on survival outcomes, as well as the recommended assessment of nutritional status using validated tools^(28)^.

In this cohort, the most common therapy received was CRT in over 90 % cases with most of the remainder receiving either definitive or adjuvant RT. Additionally, over two-thirds (79·6 %) participants evaluated for sarcopenia had P16-positive disease. The outcomes of the overall cohort with respect to survival end points is encouraging, consistent with the high prevalence of included patients with P16-positive oropharyngeal disease. In this cohort, increasing age was found to be the only variable significantly associated with sarcopenia risk, but not other pertinent clinicopathological features such as tumour stage or pre-treatment diet texture. Post-treatment sarcopenia predicted worse long-term survival when compared with those without sarcopenia post-treatment, whereas pre-treatment sarcopenia was not associated with significantly reduced survival. However, given the high prevalence of myosteatosis at both time points, no significant association with outcomes was seen. Perhaps given the very high proportion of pre-existing myosteatosis, no significant additional predictive power for outcome appeared to be evident when assessing the combined impact of post-treatment myosteatosis and sarcopenia, compared with non-sarcopenia in the same group.

Our study findings of a HR of 1·37 (95 % CI 0·63, 2·94) for worse 5-year OS with pre-treatment sarcopenia are broadly consistent with two recent meta-analyses of the association between radiologically defined sarcopenia and OS in head and neck cancer patients, where sarcopenia was found to predict worse OS^(34,35)^. Wong et al reported a higher HR of 1·98 (95 % CI 1·64, 2·39) for worse OS with sarcopenia^(35)^ and Findlay et al reported a similar HR of 2·07 (95 % CI 1·47, 2·92) for worse OS with pre-treatment sarcopenia^(34)^. As Findlay et al only included studies in their meta-analysis that evaluated sarcopenia with the gold standard method at L3 with sex-specific cut-off values^(34)^, this provides a more suitable comparison to our current study. In addition, Findlay et al found post-treatment sarcopenia was associated with worse OS (HR 2·93, 95 % CI 2·00, 4·29)^(34)^, which was comparable to our local findings (HR 2·70, 95 % CI 0·88, 6·25). These findings suggest that sarcopenia assessed at a post-treatment time point was associated with worse survival compared with pre-treatment assessment. The predictive power of post-treatment sarcopenia as compared with pre-treatment sarcopenia when predicting OS was also noted in a separate retrospective observational study performed in a similar patient group^(33)^. Taken together, these findings may further refine recommendations with respect to timing of sarcopenia assessment and prognostication. Interestingly, when OS was assessed at 1 year, there was a trend towards worse outcomes for the subset of patients who developed sarcopenia during treatment. This group was found elsewhere to account for the highest economic cost during HNSCC care in terms of unplanned admissions^(33)^, potentially suggesting that this is a subgroup with particular vulnerability to both complications of therapy and adverse outcomes, which warrants further exploration.

In this study, myosteatosis was evident at both baseline and post-treatment time points in over 90 % of participants, with the very high prevalence in the cohort preventing a meaningful statistical comparison compared with the very small non-myosteatotic group. In a separate Australian study of HNSCC patients, 63/79 (80 %) at baseline and 48/61 (79 %) post-treatment had myosteatosis, and myosteatosis at either time point was significantly associated with worse 5-year survival^(33)^. In both studies, the mean BMI of participants was in the overweight category and thus likely contributing to the high incidence of myosteatosis. Furthermore, the incidence of myosteatosis amongst HNSCC patients appears higher than sarcopenia. Given the multifactorial pathogenesis of sarcopenia, further research is required to differentiate the distinct pathological mechanisms underlying sarcopenia and myosteatosis and delineate the prognostic relevance of each.

The results of this study also indicate that sarcopenia cannot be predicted based on baseline BMI, nutritional status or proportion of weight loss during treatment but must be measured independently. Here, we have used a pragmatic approach of assessing sarcopenia radiologically from the pre- and post-treatment scans that are available as part of routine care and practice. Adopting this approach in real-world practice is likely to add value to prognostication for individual patients. Potential drawbacks from this study include that the post-treatment scan timing may have been heterogenous depending on the modality of treatment and scheduling issues, although institutional practice is reasonably standardised in this regard. Furthermore, radiological assessment of sarcopenia necessarily neglects the functional aspects of this condition, so future work could implement a more comprehensive assessment of sarcopenia as a syndrome in these patients. In this single-centre study of primarily white male participants, additional limitations in assessing survival associations relate to the generally favourable characteristics of the group leading to high rates of 5-year survival. These include a low proportion of participants with advanced disease stage (<50 % Stage III/IVA) and a high proportion with P16-positive status (75 %); all participants were treated with curative intent. Additionally, there is a limited sample size.

Importantly, it is currently unknown whether proactively treating sarcopenia or myosteatosis will influence outcomes for HNSCC patients. The predictive potential of sarcopenia in this study was more marked at the post-treatment time point compared with pre-treatment time point. A critical factor in the uptake of sarcopenia assessment in HNSCC will be determined by its acceptability and feasibility in practice, as it will require additional dedicated resources (equipment/software) as well as trained staff to undertake the assessments. Diverse cancer clinicians in Australia when surveyed had a high awareness of the importance of cancer-related malnutrition and sarcopenia, although with lower levels of knowledge relating to identification and management of these conditions; barriers in this respect included lack of knowledge and skills with respect to diagnosis and intervention^(36)^. This study further emphasises the importance of screening for sarcopenia and myosteatosis as prevalent and prognostically relevant co-morbidities in HNSCC patients as recommended by the Clinical Oncology Society of Australia’s 2021 Position Statement on Cancer-related Malnutrition and Sarcopenia^(29)^. This should be further encouraged by the recent codification of sarcopenia in the International Classification of Diseases 10th Revision (ICD-10)^(37)^. Additional future work should also be directed towards interventions which prevent or treat sarcopenia in these patients, and whether this is shown to influence outcomes.
